# Quantitative Ultrasound and Dual X-Ray Absorptiometry as Indicators of Bone Mineral Density in Young Women and Nutritional Factors Affecting It †

**DOI:** 10.3390/nu11102336

**Published:** 2019-10-01

**Authors:** Katie Schraders, Giancarla Zatta, Marlena Kruger, Jane Coad, Janet Weber, Louise Brough, Jasmine Thomson

**Affiliations:** 1 School of Food and Advanced Technology, College of Sciences, Massey University, Palmerston North 4442, New Zealand; k.schraders@massey.ac.nz (K.S.); giancarlazatta@gmail.com (G.Z.); J.Coad@massey.ac.nz (J.C.); J.L.Weber@massey.ac.nz (J.W.); L.Brough@massey.ac.nz (L.B.); 2 School of Health Sciences, College of Health, Massey University, Palmerston North 4442, New Zealand; M.C.Kruger@massey.ac.nz; 3 Fonterra Research and Development Centre, Fonterra Co-operative Group Limited, Palmerston North 4442, New Zealand

**Keywords:** quantitative ultrasound, QUS, dual X-ray absorptiometry, DXA, bone mineral density, BMD, bone mineral content, BMC, young women, calcium, protein

## Abstract

Young adulthood is an important stage in the accrual of bone mass. Young women are often unaware of the need, and how to optimize modifiable risk factors, particularly intake of nutrients associated with good bone health. In this study, an accessible way to estimate osteoporosis risk, quantitative ultrasound (QUS), is compared to the gold-standard technique dual X-ray absorptiometry (DXA) in a group of 54 healthy young women (18–26 years) from Manawatu, New Zealand, and the relationship with nutrient intake is investigated. Broadband ultrasound attenuation and speed of sound (BUA, SOS) were assessed by QUS calcaneal scans and bone mineral concentration/density (BMC/BMD) were determined by DXA scans of the lumbar spine and hip (total and femoral neck). Dietary intake of energy, protein, and calcium was estimated using three-day food diaries and questionnaires. DXA mean *Z*-scores (>−2.0) for the hip (0.19) and spine (0.2) and QUS mean *Z*-scores (>−1.0) (0.41) were within the expected ranges. DXA (BMD) and QUS (BUA, SOS) measurements were strongly correlated. Median intakes of protein and calcium were 83.7 g/day and 784 mg/day, respectively. Protein intake was adequate and, whilst median calcium intake was higher than national average, it was below the Estimated Average Requirement (EAR). No significant relationship was found between dietary intake of calcium or protein and BMD or BMC. To conclude, QUS may provide a reasonable indicator of osteoporosis risk in young women but may not be an appropriate diagnostic tool. Increased calcium intake is recommended for this group, regardless of BMD.

## 1. Introduction

Osteoporosis is a significant public health problem, with a greater prevalence in women—it affects up to 38% of women over 50 worldwide [1]. In 2007 there were 70,631 people diagnosed with osteoporosis in New Zealand, with the disease calculated to cost more than $330 million each year [2]. Osteoporosis is characterised by a reduction in bone mass and deterioration in the micro-architecture of bone tissue, which results in increased risk of fracture [3,4]. The World Health Organization (WHO) defines osteoporosis as a bone mineral density (BMD) T-score of less than −2.5 [5], except in subsets of the population where the *Z*-score is more appropriate due to being age-matched [6,7]. For premenopausal women, diagnosis of osteopenia and/or osteoporosis is not recommended, and instead patients are classified as being “below the expected range for age” if their Z-score is lower than −2.0 and “within the expected range” if above −2.0 [7].

The current “gold standard” for measuring BMD is dual X-ray absorptiometry (DXA) [8,9]. However, this method is costly, clinically-based, involves ionizing radiation, and requires a highly trained operator, adding to the complexity of using DXA as a screening tool [10]. Recent research has looked at using other methods for assessing osteoporosis risk, including quantitative ultrasound (QUS). QUS measures calcaneus bone quality by measuring the attenuation and velocity of ultrasound waves passing through the bone [11]. This method has become popular due to being low-cost and portable. It is also an ideal method for measuring young women, as it is non-ionizing [8] and is therefore safe in pregnancy. However, few studies have compared QUS to DXA in young women [8,12]. Research in post-menopausal women suggests that QUS correlates well with DXA [4,13,14], although it has also been suggested that, despite this correlation, QUS cannot accurately diagnose osteoporosis [15]. On the other hand, a recent review concluded that QUS had the potential to be a capable diagnostic tool in areas with limited access to DXA [4]. Hence, further investigation of the ability of QUS in determining the risk of osteoporosis in a young female population is warranted.

Peak bone mass (PBM) is the maximum bone density achieved in an individual’s lifespan [16]. In women, PBM is reached in the hip by 16–19 years of age and in the spine between 30 and 40 years of age [16]. Once a woman is around 24–44 years of age, bone density begins to decrease, with approximately 0.5% lost each year until menopause, when there is a significant increase in the rate of loss due to a decline in oestrogen production [17]. Many factors affect the accrual of PBM, such as genetic (accounts for 40–80% of the variance) and environmental (including diet, physical activity, and body mass) [16] factors. Women face challenges optimizing the modifiable risk factors, including inadequate nutrient intake due to dieting and restrictive eating [18]. Young women, who have not completed bone mineral deposition, often fail to meet dietary recommendations, particularly for calcium [19]. Whilst young women in developed countries are likely to meet their protein requirements [19], it is important that they do, as a recent study indicated protein intake has a beneficial effect on bone mass when calcium intake is also adequate [20].

Therefore, the aim of the study was to investigate both how QUS compares to DXA in a population of young women of reproductive age, and their intake of protein and calcium, at an age when their PBM could still be increased. 

## 2. Materials and Methods

### 2.1. Study Design

This cross-sectional study was undertaken at Massey University, Palmerston North campus, from May to November 2017. Participants visited the Human Nutrition Research Unit, on campus, where they completed questionnaires regarding their current lifestyle, and knowledge and beliefs about bone health, and had BMD and bone quality measured by DXA and QUS, respectively. Participants were provided with a three-day diet diary to complete at home. The outcome measures reported in the current article include the assessment of bone parameters using DXA and QUS, and intake of nutrients associated with bone health, described below. 

### 2.2. Participants

Ninety-nine healthy women from the Manawatu and surrounding regions, aged 18 to 26 years, were recruited. The following exclusion criteria were applied: diagnosis of Juvenile Rheumatoid Arthritis, Type 1 diabetes, Osteogenesis Imperfecta, uncontrolled thyroid disease, inflammatory bowel disease, chronic renal disease, and clinically significant liver disease; medical treatment with daily corticosteroid tablets for greater than three months; any other condition affecting bones and/or absorption of nutrients. All participants were fully informed about the requirements of the study and gave written consent. The study was approved by the Massey University Human Ethics Committee (Southern A), Reference Number SOA17/06. 

### 2.3. Dietary Analysis

Participants were requested to provide a three-day estimated food record. They were asked to record all food and beverage intake over two weekdays and one weekend day and were requested to be as accurate as possible, to use household measures to estimate intakes, and to provide any recipes used. All food diaries were examined for completeness and then discussed with participants in a face-to-face interview if there were suspected entry errors or missing values. Dietary data were analysed using Foodworks 9 (Xyris Software Pty, Brisbane, Australia) to estimate calcium, protein, and energy intakes. 

### 2.4. Bone Densitometry

DXA scans of the lumbar spine and hip (total and femoral neck) were carried out by a qualified operator using a Hologic QDR-Discovery A densitometer (Hologic Inc., Bedford, MA, USA) to determine bone mineral content (BMC, grams), bone mineral density (BMD) (grams/centimetre^2^), and *Z*-scores. The DXA machine was calibrated daily with a spine phantom according to the manufacturer’s instructions. Precision was determined by calculating the coefficient of variation, which was within 0.45–0.54% for all measurements. 

### 2.5. Bone Quality

Bone quality was measured using an Achilles QUS ultrasonometer (Lunar Achilles Insight, GE Lunar Corporation Inc., Madison, WI, USA). Bone quality was measured in the non-dominant foot to reduce variability. The machine was calibrated daily according to the manufacturer’s instructions. Stiffness index (SI) was calculated from the speed of sound (SOS) and broadband ultrasound attenuation (BUA) and the *Z*-scores were determined [21]. 

### 2.6. Statistical Analysis

Statistical Analysis Software (SAS) (Version 9.4) (SAS Institute Inc., Cary, NC, USA) was used for all analyses. All variables were checked using the Kolmogorov–Smirnov test for normal distribution prior to analysis. Normally distributed data were reported as the mean ± the standard deviation (SD). The ability of dietary calcium and protein to predict BMD (g/cm^2^) and BMC was investigated using multiple linear regression analysis. The relationship between QUS and DXA was explored using univariate linear regression. Statistical significance was set at *p* < 0.05.

This study is a subset of a larger observational study. Sample size to assess the correlation of DXA and QUS was calculated [22] based on the predicted population of 18–25-year-old females (267,100 in New Zealand) in 2017 [23]. Assuming a 90% confidence level, margin of error of 10%, and a variability of 10%, the minimum sample size needed was 35 women. Factoring in for incomplete data sets/drop out between visits of 30%, a sample size of 50 women was required to assess the correlation between these measurement methods.

## 3. Results

### 3.1. Demographic

Of the 99 participants who took part, 60 completed a three-day food diary, however, two participants’ results were not included in this analysis due to insufficient information from their three-day food diary. Sixty women had a DXA scan, however, four were excluded; two due to equipment malfunction and two due to problems with participant body positioning during the scan. A total of 54 participants successfully completed all aspects of the study. The characteristics of these participants can be seen in Table 1. 

### 3.2. Nutrient Intake

The average intake of calcium was 829 mg in participants 19 years and older (*n* = 47) and 927 mg in 18-year-old participants (*n* = 7). Three 18-year-old participants met the estimated average requirement (EAR) for calcium, while 44.7% of participants (21/47) aged 19 years and older met this recommendation. Study population averages are represented in Table 2.

The average intake of protein for participants 19 years and older was 88.9 g/day, compared with 75.7 g in 18-year-old participants. When factoring in the weight of these participants to compare intake with EAR, the average protein intake was 1.4 g/kg for participants 19 years and older and 1.07 g/kg for the 18-year old participants. All participants met the EAR for protein intake.

Participants were questioned about their use of supplements. Although no participants reported deliberately supplementing with any key nutrients specifically for bone health, 11% reported taking a woman’s multivitamin preparation. Protein supplementation was also reported by 11% of participants. 

The validity of participants’ dietary intake was estimated using the Goldberg cut-offs [24]. Under- and over-reporters were estimated using a physical activity level of 1.55 [24]. This suggests that 7/54 under-reported and 2/54 over-reported their intake.

### 3.3. Bone Health

The mean Z-score for all sites was within the expected range (*Z*-score of >−2.0) [7] (see Table 3). The DXA measurements identified four people defined as having BMD below the expected range for age in their lumbar spine according to the International Society for Clinical Densitometry (ISCD) cut-offs (*Z*-score of <−2.0) [7]. One participant was also found to have BMD below the expected range for age in the hip (*Z*-score of <−2.0). Of these five participants, only one was considered at risk, assessed by QUS with a *Z*-score of less than −1.0 [12].

There was no relationship between BMC or BMD and age. There was a significant positive correlation between weight and hip BMD (*R* = 0.595, *P* < 0.0001).

### 3.4. Evaluation of QUS

A significant positive correlation was shown between DXA and QUS (*P*-value < 0.05), see Table 4. However, the low correlation coefficients suggest that a linear regression is not the best fit for this relationship. Although the relationship is strong, this would therefore not be a good model to predict an individual’s hip BMD based on QUS (or vice versa). The stiffness index, calculated from BUA and SOS, correlated better with hip BMD than SOS or BUA independently (*R* = 0.646, *P* < 0.0001). 

### 3.5. Nutrition and Bone Health

No significant relationship was found between the dietary intake of calcium or protein and BMD or BMC of the hip (*P* > 0.05). There was also no significant relationship found between calcium or protein intake and the BMD of the lumbar spine (*P* > 0.05). A modest but statistically significant relationship was found between calcium intake and BMC of the lumbar spine (*P* < 0.05). 

## 4. Discussion

The mean Z-score for all sites measured fell within the expected range for participants of this age (>−2.0) [7]. When comparing the BMD of participants in this study to that found in similar studies of young women of the same age range, hip BMD values were similar [25,26]. However, the current study found lower lumbar spine BMD (>1 standard deviation) than three of four comparable studies [26,27,28]. The findings of this study support previous research which found a strong relationship between BMD and QUS [4,8,12,13,14]. However, it should be noted that of the five participants identified as having BMD below the expected range for age [7] (four identified in the lumbar spine and one in the hip), only one of these (with a low *Z*-score for lumbar spine) was considered at risk based on the QUS *Z*-score [12]. The QUS also identified a further five people at risk of osteoporosis (*Z*-score <−1) [12], whose BMD for all sites was within normal range (*Z*-score >−2) when assessed by DXA [7]. The QUS measures bone quality of the calcaneus, which is predominantly trabecular bone, and it is therefore considered to be representative of the trabecular bone in the hip [29] and may not be a good representation of BMD in the lumbar spine. This finding is of concern, since authors of a recent review concluded that QUS could be used as a standalone screening method [4]; results of the current study suggest that those at the greatest risk were not identified using this method. To test this further a population needs to be recruited with a greater number of young women at higher risk of osteoporosis.

The median intake of calcium was greater than the intake reported for 19–30-year-old women in the most recent New Zealand Adult Nutrition Survey [19]. However, this intake is below the EAR of 840 mg per day for women 19–30 years of age and 1050 mg for those aged 18-years [30]. Comparable studies in other countries reported similar findings of low calcium intakes in young women [25,26,28]. Reasons for poor calcium intakes in this group may be due to limited knowledge of the beneficial role of calcium for bone health in young adulthood [31,32], ethical considerations related to the dairy industry and farming practices [32], the perceived high cost of dairy products [32,33] resulting in calcium-rich foods being less accessible for students, the perception of dairy foods as fattening [34], and that this population group commonly practices restrictive eating, which might result in reduced consumption of dairy foods, affecting calcium intake. Some limitations of the dietary analysis should be noted. Whilst no participant reported taking calcium-only supplements, some took multivitamin preparations, and because insufficient information regarding the type of multivitamin was provided, it was not possible to determine calcium coming from supplements, so the analysis of calcium intake was from food only. All participants met the EAR for protein intake. Intakes were higher than found previously for this age group in both the New Zealand Adult Nutrition Survey [19] and the most recent evaluation of university student diets in New Zealand [35]. The reason for the relatively high protein intake in this study is unclear. Nonetheless, protein intake at this level is unlikely to pose a risk to bone health. Whilst early studies suggested a diet high in protein would result in increased bone resorption [36], a recent systematic review and meta-analysis reported that similar intakes of protein had no detrimental effect on bone health [37]. 

The median energy intake for the study participants was 8245 kJ per day, which was similar to that seen in women in this age group in the New Zealand Adult Nutrition Survey [19]. Under or over-reporting is a concern in dietary assessment, however the 13% of possible “under-reporters” in this study (energy intake below calculated expenditure) were considered to have plausible intakes, since research suggests that tertiary students often have poor quality diets [38], experience food insecurity [39,40,41], and that restrictive eating is commonly practised in this age group [42]. Therefore, it was decided not to exclude suspected “under-reporters” from the study [43]. The current study estimated that 4% were over-reporters, which is consistent with that previously reported [24]. However, one of the two “over-reporters” was known to be an athlete, so using 1.55 Physical Activity Level (PAL) for the Goldberg cutoff was inappropriate [24]; therefore, it was decided not to exclude any suspected “over-reporters” from the dataset either. 

No significant relationship was found between dietary intake of calcium and BMD or BMC in the current study. This is not surprising given that no confounding variables were included in the analysis, and dietary intake for this study was collected via a three-day food diary, so it provided only a snapshot of calcium intake at the time of the study and, as discussed previously, it was not possible to determine calcium content from multivitamin intake. Long-term calcium intake has been positively linked with BMD [44]; however, the three-day food diary may not have been representative of participants’ long-term habitual calcium intake. Calcium intake and dairy intake have long been considered to be beneficial to bone health, with numerous studies reporting positive associations between their consumption and bone mass [27,45,46]. However, recent controversy about the relationship between calcium and milk consumption and their impact on fracture risk [47] suggests both dietary sources of calcium intake and other influencing factors should be investigated over longer time periods; ideally, participants in the current study would be followed up in 10- and 30-years’ time. Based on current recommendations, it is appropriate to continue promoting calcium intake for this age group, regardless of current BMD. 

No significant relationship was found between the dietary intake of protein and BMD or BMC in the current study. Vatanparast and colleagues [20] found protein intake was positively associated with BMD in young women with adequate calcium intake [20]. Calcium absorption and excretion are affected by a range of other nutrients’ bioavailability, and poor calcium intake can be exacerbated by poor or imbalanced intake of other nutrients [48]. Therefore, investigation of the relationship between BMD and nutrients should consider more nutrients. One of the limitations of this study was that phosphate intake was not analysed; the calcium:phosphate ratio is important for bone mineralization [47]. Owing to undetermined/missing phosphate values in the Foodworks™ database, this intake cannot be reported. It was also not possible to determine the influence of vitamin K and vitamin D [49] status because of the financial limitations of the study. 

Further investigation into dietary factors with negative effects on bone health, for example, the impact of soft drinks on BMD, is warranted. A recent research study by Hammad and Benajiba [50] identified the negative correlation between soft drink consumption and BMD to be the most significant dietary interaction. As well as soft drinks contributing phosphoric acid and caffeine to the diet, which are associated with poorer bone health [51], emerging evidence from animal studies and a few small human studies suggests that both caloric and noncaloric sweeteners in soft drinks may negatively affect bone [52]. Individuals living in New Zealand have the highest intake of sugar of any OECD country [53], with 39.6% of 15–18-year-old and 27.8% 19–30-year-old women consuming more than three soft/energy drinks each week [19]. New Zealand beverages have also recently been reported to have greater sugar levels per 100 mL than comparable drinks in Australia, Canada, and the UK [53]. It is therefore recommended that further research investigate the relationship of dietary factors reported to have negative effects on bone health in more depth and over longer periods of time. 

Limitations of this study include the small sample size and possible recruitment bias. Due to the location and recruitment at university and polytechnic campuses, this sample population is unlikely to be representative of this age group. Participants in this study have a greater level of education and probably more positive socioeconomic factors, which are known to influence chronic diseases such as osteoporosis [54]. This may mean that the study population is less likely to be vulnerable to poor bone health than the general population. One of the most significant limitations was the reliance on self-reported dietary intake, as this may have resulted in participants selectively under-reporting food which is perceived to be unhealthy [33]; this is always a limitation in this type of study. Whilst this was a fairly homogenous population of healthy (as conditions and medications affecting bone mass were screened out) young women between 18 and 25 years of age, a further limitation is that confounders such as smoking and physical activity were not evaluated or included in the multi-variate regression model.

There are also challenges in the clinical interpretation of BMD or QUS results in premenopausal women. In postmenopausal women, the relationship between low BMD and prediction of fracture risk is much clearer. Low trauma fractures are much less likely in younger women but are an indicator of future fracture risk and associated poor bone quality [55]. Routine screening of BMD by DXA scans is not recommended in younger women, and a low BMD *Z*-score (comparison to an age-matched reference population) in the absence of a low trauma fracture is interpreted as idiopathic low bone density (“below the expected range for age”) by the Internal Society of Clinical Densitometry (ISCD), rather than being regarded as the diagnostic criterion for osteoporosis as it is in postmenopausal women [55]. Part of the reason for this recommendation is that the low incidence of abnormal results does not justify the cost of DXA scans and there is a resistance to exposing women of reproductive age to ionising radiation. There is a need for adequately powered prospective studies to identify whether low hip BMD in young women is a valid predictor of bone health and future fracture risk. The ability to identify young women at increased risk of later poor bone health, who may still be actively accruing bone mass, could offer the opportunity to design targeted public health strategies aimed at promoting osteoprotective behaviour to either increase bone mass or to abrogate its loss.

## 5. Conclusions

The findings of this study suggest that QUS may be a useful tool for assessing bone health in young women, but data from this study, which focused on young healthy women, cannot confirm whether it is an appropriate diagnostic or screening tool in this age group. The study found that QUS correlated with hip BMD in this population, suggesting that QUS may be a useful tool in community health promotion and research, even if it is not found to be suitable for determining an individual’s risk of osteoporosis. Intake of calcium and protein were not correlated with BMD, and although calcium intake was greater than the national average in the last national nutrition survey, it was still below the EAR in this group. This suggests that it is important to identify the barriers limiting adequate calcium intake for all low consumers, not just those with low BMD. 

## Figures and Tables

**Table 1:** Characteristics of the sample population.

Parameters	Participants (*n* = 54)	Range
Age (years)	20 (19, 22) *	18–26
Height (cm)	167.8 ± 6.2 †	151.5–181.2
Weight (kg)	66.2 ± 9.3 †	49.7–93
BMI (kg/m^2^)	23.5 ± 3.0 †	18.4–33.8

* Median (25th percentile, 75th percentile); † Mean ± SD; BMI, Body Mass Index.

**Table 2:** Dietary intake of calcium and protein.

Nutrient	Study Group (*n* = 54)	NZ Women ^1^	Nutrient Reference Values (NRVs)	
			EAR ^2^	
			14–18 Years	19–30 Years
Calcium (mg)	784 (659, 976) *	704 *	1050	840
Protein (g)	83.7 (72.0, 101.4) *	72 *	35 g/day (0.62 g/kg)	37 g/day (0.60 g/kg)
Protein (%EI)	18.7† ± 5.6	15.4 †	18.4–33.8	
Energy kJ	8245 (6817, 9482) *	7448 *		

* Median (25th percentile, 75th percentile); † Mean ± SD. NZ, New Zealand; NRVs, Nutrient Reference Values; EAR, estimated average requirement; EI, energy intake; kJ, kilojoule.^1^ University of Otago and Ministry of Health. 2011. A Focus on Nutrition: Key findings of the 2008/09 New Zealand Adult Nutrition Survey. Wellington: Ministry of Health. ^2^ National Health and Medical Research Council, Australian Government Department of Health and Ageing, New Zealand Ministry of Health. Nutrient Reference Values for Australia and New Zealand. Canberra: National Health and Medical Research Council; 2006.

**Table 3:** Summary of bone parameters.

		BMD (g/cm^2^)	BMC	*Z*-Score	BUA	SOS
Heel				0.41 ± 1.08†	177.61 ± 12.81†	1598.29 ± 40.01†
					(94.1–149.3)	(1516.6–1700.9)
Hip	Total	0.97 ± 0.15†	32.38 ± 6.25†	0.19 ± 1.199†		
		(0.63–1.35)	(19.52–51.20)	(−2.6–3.4)		
	FN	0.86 ± 0.14†				
		(0.57–1.15)				
Spine		1.00 ± 0.12†	59.56 ± 11.37†	−0.20 ± 1.13†		
		(0.77–1.36)	(38.03–90.60)	(−2.4–2.9)		

† Mean ± SD (Range); BMD, bone mineral density; BMC, bone mineral content; BUA, broadband ultrasound attenuation; SOS, speed of sound; FN, femoral neck.

**Table 4:** Correlation between QUS and DXA.

	Linear Regression (R^2^)	Correlation Coefficient (R)	*P*-Value for Correlation
BUA versus Hip BMD	0.340	0.583	<0.0001
BUA versus Lumbar spine BMD	0.357	0.597	<0.0001
BUA versus Femoral Neck BMD	0.247	0.497	0.0002
SOS versus Hip BMD	0.291	0.539	<0.0001
SOS versus Lumbar spine BMD	0.134	0.366	0.0007
SOS versus Femoral Neck BMD	0.230	0.479	0.0003

*N* = 53 due to no SOS results for one participant; BUA, broadband ultrasound attenuation; SOS, speed of sound.
